# Disulfiram attenuates hypoxia-induced pulmonary hypertension by inhibiting GSDMD cleavage and pyroptosis in HPASMCs

**DOI:** 10.1186/s12931-022-02279-0

**Published:** 2022-12-16

**Authors:** Shunlian Hu, Lu Wang, Yahan Xu, Fajiu Li, Tao Wang

**Affiliations:** 1 Department of Respiratory and Critical Care Medicine, Tongji Hospital, Tongji Medical College, Huazhong University of Science and Technology, Wuhan, 430030 People’s Republic of China; 2 The Center for Biomedical Research, National Health Committee (NHC) Key Laboratory of Respiratory Disease, Tongji Hospital, Tongji Medical College, Huazhong University of Science and Technology, Wuhan, People’s Republic of China; 3 Department of Respiratory and Critical Care Medicine, Miyun Teaching Hospital of Capital Medical University, Beijing, People’s Republic of China; 4 Department of Respiratory and Critical Care Medicine, Peking University First Hospital, Miyun District, Beijing, People’s Republic of China; 5 Department of Respiratory and Critical Care Medicine, Beijing Miyun Hospital, Beijing, People’s Republic of China; 6 The Sixth Hospital of Wuhan City, Affiliated Hospital of Jianghan University, Beijing, People’s Republic of China

**Keywords:** Pulmonary hypertension, Pyroptosis, Disulfiram

## Abstract

**Background:**

Pulmonary hypertension (PH) is characterized by progressive pulmonary arterial remodelling, associated with different severities of inflammation and altered immune processes. Disulfiram eliminates the formation of N-gasdermin D (GSDMD) plasma membrane pores to prevent pyroptosis. Pyroptosis is a form of lytic cell death characterized by inflammasome activation and proinflammatory cytokine release that acts in the development of PH. We sought to investigate whether disulfiram could alleviate hypoxia-induced PH by inhibiting pyroptosis.

**Methods:**

To investigate whether disulfiram alleviates the progression of pulmonary hypertension, rodents were exposed to chronic hypoxia (10% oxygen, 4 weeks) to induce PH. The severity of PH was assessed by measuring right ventricular systolic pressure, mean pulmonary artery pressure, and the degree of right ventricular hypertrophy. Western blotting was used to measure proteins associated with the pyroptosis pathway, and ELISA was performed to measure the secretion of IL-18 and IL-1β, both of which are the primary methods for assessing pyroptosis.

**Results:**

IL-18 and IL-1β concentrations were higher in patients with PH than in normal controls. Disulfiram suppressed the progression of PH in mice and rats through the alleviation of pulmonary arterial remodelling. Pyroptosis-related proteins and the inflammasome were activated in rodent models of PH. Disulfiram inhibited the processing of GSDMD into N-GSDMD and attenuated the secretion of IL-1β and IL18. In vivo experiments showed that disulfiram also inhibited lytic death in HPASMCs.

**Conclusions:**

Disulfiram treatment reduces PH progression through suppressing vascular remodelling by inhibiting GSDMD cleavage and pyroptosis. It might become a novel therapeutic option for the treatment of PH.

**Supplementary Information:**

The online version contains supplementary material available at 10.1186/s12931-022-02279-0.

## Background

Pulmonary hypertension (PH) is a class of haemdynamic disturbances characterized by progressive pulmonary arterial remodelling, vasoconstriction and thrombosis associated with different severities of inflammation and altered immune processes, ultimately leading to right heart failure and death [1]. Hypertrophy and proliferation of human pulmonary artery smooth muscle cells (HPASMCs) are considered important pathophysiological changes of pulmonary arterial remodelling in PH [2]. Although inflammasome activation in pulmonary vessels has been considered a critical factor for pulmonary arterial remodelling [3], there are no specific therapeutic targets.

Pyroptosis is a form of inflammatory cell death different from apoptosis, necrosis, ferroptosis, and autophagic cell death, characterized by the activation of the inflammasome and the caspase family and the release of numerous proinflammatory cytokines [4]. Pyroptosis is broadly classified into classical caspase-1-dependent cell pyroptosis and nonclassical caspase-4/5/11-dependent cell pyroptosis. Inflammasome complexes, caspase1, the gasdermin family, interleukin- 18 (IL-18) and IL-1β are pivotal components of caspase-1-dependent pyroptosis [5]. Activated inflammasomes, through such components as such as nucleotide-binding domains and leucine-rich repeat receptors, recruit apoptosis-associated speck-like protein containing a caspase-recruitment domain (ASC) to assemble into inflammasome complexes to engage caspase-1 activation [6]. Among the various inflammasome complexes, the nucleotide-binding oligomerization domain-like protein 3 (NLRP3)/ASC complex is the most well characterized [7]. The NLRP3/ASC complex activates caspase-1 to the CI. The caspase-1 fragment causes cleavage of the gasdermin D (GSDMD), pro-IL-18 and pro-IL-1β, leading to the formation of cell membrane pores and the release of IL-1β and IL-18 [8]. Pyroptosis is involved in the development and progression of PH. HPASMC pyroptosis contributes to PH [9–11]. Inhibition of the NLRP3 pathway attenuates LPS-induced acute heart failure in monocrotaline-induced PH rats [12]. The absence of ASC has a protective effect in hypoxic PH [13, 14]. Knockout of caspase-1 attenuates the pathogenic features of PH, such as pulmonary arterial remodelling, right heart dysfunction, and pulmonary vascular fibrosis [15]. IL-1β and IL-18 also drive the apoptosis resistance and overproliferation of HPASMCs through several different mechanisms [15–18]. These results indicate that pyroptosis plays a significant role in the occurrence and progression of PH.

Disulfiram (DSF), an FDA-approved drug for alcohol addiction, has recently been shown to block pyroptosis and cytokine release in cells by inhibiting the binding of N-GSDMD to acidic phospholipids in the inner leaflet of the cell plasma membrane [19]. Though existing treatments for PH have significantly improved the outcomes of patients with PH [20], many patients do not meet the expectations. In this study, we investigated whether DSF could alleviate right ventricular systolic pressure (RVSP), right ventricular hypertrophy (RVH) and vascular remodelling in rodent models of hypoxia-induced PH by inhibiting the pyroptosis of HPASMCs, providing a novel therapeutic option for the treatment of PH.

## Materials and methods

### Measurements of plasma IL-1β and IL-18 in PH patients

The blood samples used in this study were obtained from inpatients who underwent right heart catheterization in Wuhan Sixth Hospital from 2020 to 2022 (Additional file 1: Table S1). Fourteen patients with idiopathic, hereditary, pulmonary disease-associated and left heart-associated PH and 16 patients without PH were selected for this study. Pulmonary artery pressure was measured in all patients by right heart catheterization. The diagnostic criterion for PH was a mean pulmonary artery pressure at rest (mPAP) ≥ 25 mmHg measured by right heart catheter [2].

### Cell culture

HPASMCs were obtained from iCell Bioscience (Shanghai, China). Cells were cultured in Dulbecco’s modified Eagle medium (DMEM) (Keygen, China) supplemented with 10% foetal bovine serum (ProCell, China) and smooth muscle cell growth supplement (#1052, ScienCell) at 37 °C in a 5% CO_2_ incubator. Hypoxia was induced by growth in a hypoxic incubator at 2% O_2_.

### Animals and treatments

Male animals were selected in this study to avoid hormonal effects. Animals were marked with a numerical code to ensure that they were randomly assigned to a different group, as follows: (a) normoxia for 28 days followed by vehicle (corn oil), (b) normoxia for 28 days followed by DSF (HY-B0240, MCE, Shanghai, China), (c) hypoxia for 28 days followed by vehicle (corn oil), and (d) hypoxia for 28 days followed by DSF. In the hypoxia group, male C57 mice and Sprague–Dawley rats were continuously exposed to hypoxia (10% O_2_) in a normobaric hypoxic chamber for 4 weeks. The hypoxic environment was achieved by supplementing with 100% nitrogen and was checked with an oxygen meter (CY-12C, Hangzhou, China) to detect the oxygen concentration. The normoxia group was in the same room but with 21% O_2_. Then each control group received vehicle (corn oil) and the each DSF group received DSF through intragastric administration at a dose of 50 mg/kg/day after 28 days of hypoxia.

### Haemodynamic analysis and ventricular weight measurement

After the mice were anaesthetized, they were intubated and ventilated with a small animal ventilator (DW3000-B, Huaibei Zhenghua Biological Instrument Equipment Co., Ltd., Anhui, China) with a tidal volume of 1 ml and a respiratory frequency of 100 breaths/min using room air. RVSP proceeded immediately after opening the chest. Specifically, a needle with 0.45 mm in diameter and 16 mm in length was carefully inserted into the apex of the right ventricle.

After the rats were anaesthetized, the right jugular vein was exposed, and a PE-50 curved catheter was inserted into the right jugular vein to record the mean pulmonary artery pressure (mPAP). All animals were anaesthetized by intraperitoneal injection of sodium pentobarbital (30 mg/kg). The RVSP and mPAP were recorded using a PowerLab data acquisition system (AD instrument) and averaged over at least 10 consecutive heartbeats.

At the end of the haemodynamic measurements, the venous blood, heart and lavaged lungs were collected from the animals. The blood was centrifuged at 2500 rpm for 10 min, and the plasma was collected and stored at − 80 °C. The heart was taken to measure the right ventricular hypertrophy index, also referred to as the Fulton index [(the weight ratio of the wall of the right ventricle to the left ventricle plus septum: RV/(LV + S)]. The right lung was frozen in liquid nitrogen, stored at − 80 °C and used for subsequent western blot (WB) experiments. The left lung was fixed with 4% paraformaldehyde at room temperature (r.t.) for subsequent paraffin embedding.

### Histological analysis

Fixed left lungs were sectioned at the largest cross-section (5 μm) and subjected to haematoxylin and eosin (HE) staining. Histological snapshots were taken under a microscope (Olympus, Tokyo, Japan). Distal pulmonary arteries with a diameter of 50–100 μm were selected for taking histological snapshots under a microscope (Olympus, Tokyo, Japan). The extent of pulmonary artery remodelling was quantified by calculating the ratio of the inner wall area to the maximum area of the vessel.

### Immunofluorescence staining

The paraffin-embedded lung tissue sections were baked at 65 °C for 45 min and deparaffinized. Antigen repair was conducted using the microwave thermal repair method. BSA (10%) was used to block nonspecific antigens for 45 min at r.t. The sections were then incubated overnight at 4 °C with the following primary antibodies: α-SMA (1:100, Proteintech). Next, the sections were incubated with the secondary antibody corresponding to the primary antibody for 1 h at 37 °C. Finally, the nucleus was counterstained with DAPI for 10 min. The sections were sealed with anti-fluorescence quencher and stored at 4 °C in the dark. Fluorescence microscopy was performed on an Olympus fluorescence microscope. The fluorescence intensity was quantified using ImageJ software.

### Western blot analysis

Total protein was extracted from lung tissues and cells into RIPA buffer through ultrasonic lysis, and then the total protein concentration was measured using a BCA assay kit (Beyotime, Shanghai, China). Standard 10% or 12.5% sodium dodecyl sulfate–polyacrylamide gel electrophoresis (SDS-PAGE) was used to separate proteins of different molecular weights. The proteins were electrotransferred to polyvinylidene difluoride (PVDF; Millipore, USA) membranes by electrophoresis. The membranes were blocked with Tris-buffered saline with Tween 20 (TBST) containing 5% nonfat skimmed milk at r.t. for 1 h. The primary antibody was incubated at 4 °C, and the secondary antibody was incubated at r.t. The blots were visualized with chemiluminescent reagents (Proteintech, China). Semiquantitative analysis was conducted with ImageJ software. The following primary antibodies were used: total and cleaved N-terminal GSDMD antibody (TA4012), NLRP3 antibody (T55651), IL1 beta antibody (TA5103) were purchased from Abmart (Shanghai, China). GSDMD Full Length + N-terminal (A10164), caspase-1 (A16792), ASC (A1170), IL18 (A20473) antibodies were purchased from ABclonal (Wuhan, China).

### ELISA

ELISA kits for mouse IL-1β (RK00006), human IL-1β (RK00001), rat IL-1β (RK00009) and human IL-18 (RK00176) were obtained from ABclonal (Wuhan, China), and mouse IL-18 (EK0433) and rat IL-18 (EK0592) were obtained from BOSTER (Wuhan, China). ELISA was performed according to the manufacturer’s instructions.

### Hoechst 33,342 and propidium iodide (PI) fluorescence staining

Cell death was assessed by Hoechst 33,342 and PI fluorescence staining according to the manufacturer’s instructions (c1056, Beyotime, China). Briefly, HPASMCs were seeded in 6-well plates and treated with DSF (10 μmol/L) for 24 h with or without hypoxia (2% O_2_). The cell culture supernatant was removed, and cell staining buffer containing 5 μL each of Hoechst 33,342 and PI was added. Then the cells were incubated for 25 min at 4 °C in the dark. Stained cells were checked under an inverted fluorescence microscope (OLYMPUS IX71). The percentage of PI-positive cells in each field was recorded and analysed using ImageJ software.

### Lactate dehydrogenase release assay

LDH release was detected with a lactate dehydrogenase cytotoxicity assay kit (C0017, Beyotime, China). Briefly, HPASMCs were cultured in 96-well plates until the cells proliferated to approximately 60% confluence and then were treated with DSF (10 μmol/L) for 24 h with or without hypoxia. The cell culture supernatant (120 μL) was collected and mixed with 60 μL of substrate and then incubated for 30 min at r.t. The OD490 was measured with a full-wavelength microplate reader (Thermo Fisher).

### Statistical analysis

For animal experiments, “N” represents the number of animals in which the same treatment was performed; for cell experiments, “N” represents the number of independent experiments performed with primary cells from different individuals or cells at different passages. All data are expressed as the mean ± SEM. The data were analysed for statistical significance with IBM SPSS Statistics software (version 23). Graphs were generated with GraphPad Prism 8.0 (GraphPad Software, USA). The differences were analysed using Student’s t test or one-way ANOVA followed by Bonferroni’s multiple comparison test when the data followed a Gaussian distribution. Nonparametric tests (Kruskal–Wallis test) were carried out when data were not normally distributed. The experiments followed the principle of randomization, and the data analysis was performed in a blinded manner whenever possible. P < 0.05 was considered statistically significant.

## Results

### Lung tissue pyroptosis-related gene expression and plasma IL-1β and IL-18 concentration in patients with PH increase

**Fig. 1:**
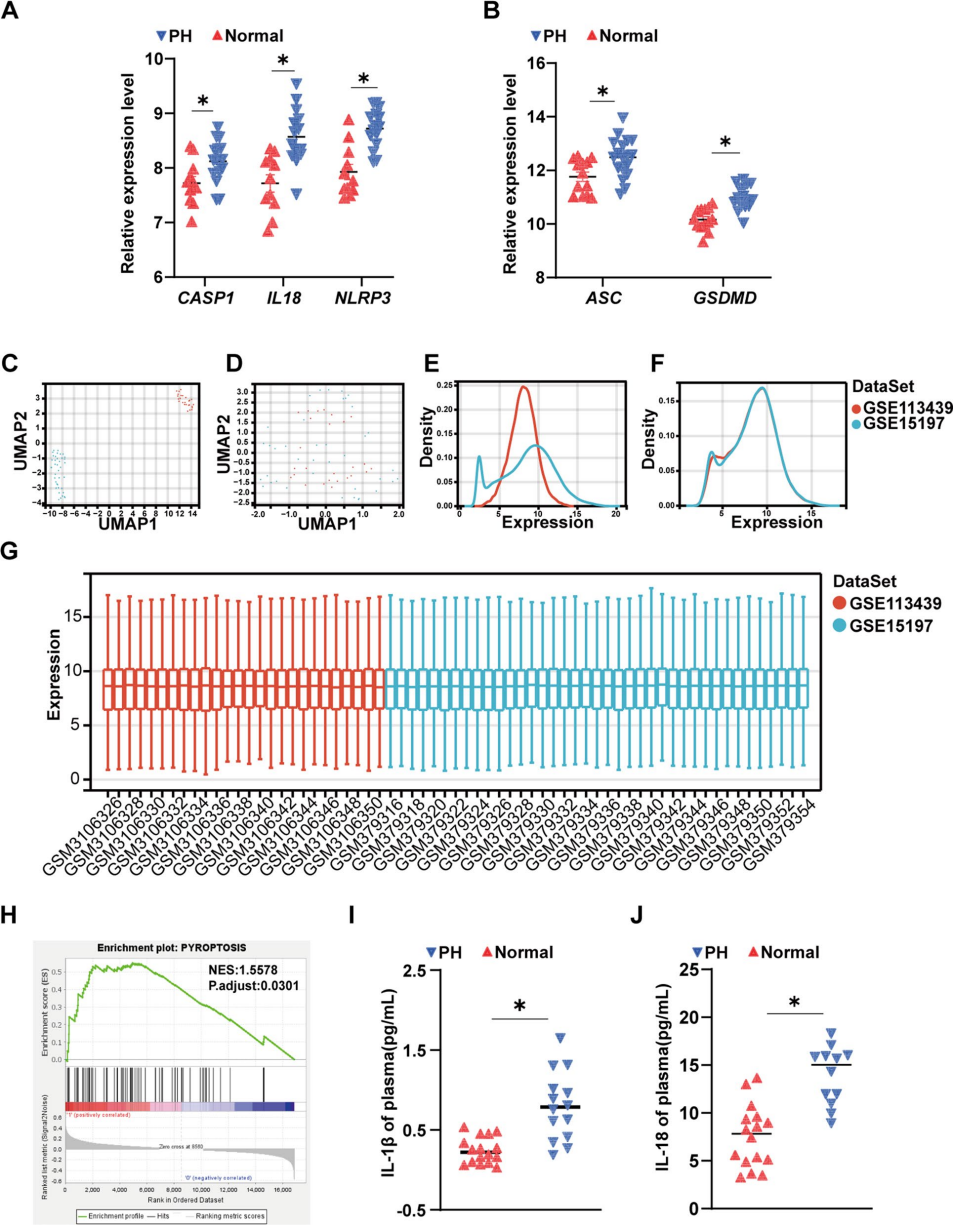
Patients with pulmonary hypertension (PH) have high expression levels of canonical caspase-1-dependent pyroptosis genes. **A** The gene expression levels of *NLRP3*, *IL18* and *CASP1* in the lung tissue of patients with PH (n = 15) and control subjects (n = 11). The database is GSE113439. **B** The gene expression levels of *ASC* and *GSDMD* in the lung tissue of patients with PH (n = 18) and control subjects (n = 13). The database is GSE15197. **C** An unnormalized UMAP plot of GSE113439 and GSE15197. **D** A normalized UMAP plot of the GSE113439 and GSE15197. **E** An unnormalized expression density plot of GSE113439 and GSE15197. **F** A normalization expression density plot for GSE113439 and GSE15197. **G** Normalized expression distribution plots for GSE113439 and GSE15197. **H** GSEA of the pyroptosis gene set. Normalized enrichment score (NES) = 1.5578, nominal P value = 0.0301. **I** Plasma IL-1β levels in patients with PH increase. **J** Plasma IL-18 levels in patients with PH increase. Values are the mean ± S.E.M. and were analysed by the unpaired two-tailed Student’s t test. *P < 0.05 vs. the normal control

We downloaded and re-analysed public databases GSE113439 and GSE15197 [21, 22] and observed that the expression levels of *NLRP3, ASC (PYCARD), IL18, CASP1* and *GSDMD* were higher in patients with PH than normal controls (Fig. 1A and B). GSE113439 and GSE15197 were merged after removing batch effects [23]. We integrated GSE113439 and GSE15197, and unified modal approximation and projection (UMAP) analysis and expression density plots revealed the spread of the respective datasets before and after the removal of batch effects (Fig. 1C–F) [23]. We then normalized the expression profiles of these 57 samples (Fig. 1G). Gene set enrichment analysis (GSEA) of pyroptosis-related genes was performed on the integrated dataset (Fig. 1H). The genes related to pyroptosis were searched in the PubMed database (https://pubmed.ncbi.nlm.nih.gov/) and the Gene Ontology Resource (http://geneontology.org/). Finally, 67 genes were collected (Additional file 1: Table S2) [24, 25]. In addition, we observed that the levels of IL-18 and IL-1β were significantly higher in PH patients than in the control group (Fig. 1I and J).

### DSF attenuates the progression of pulmonary hypertension in mice

**Fig. 2:**
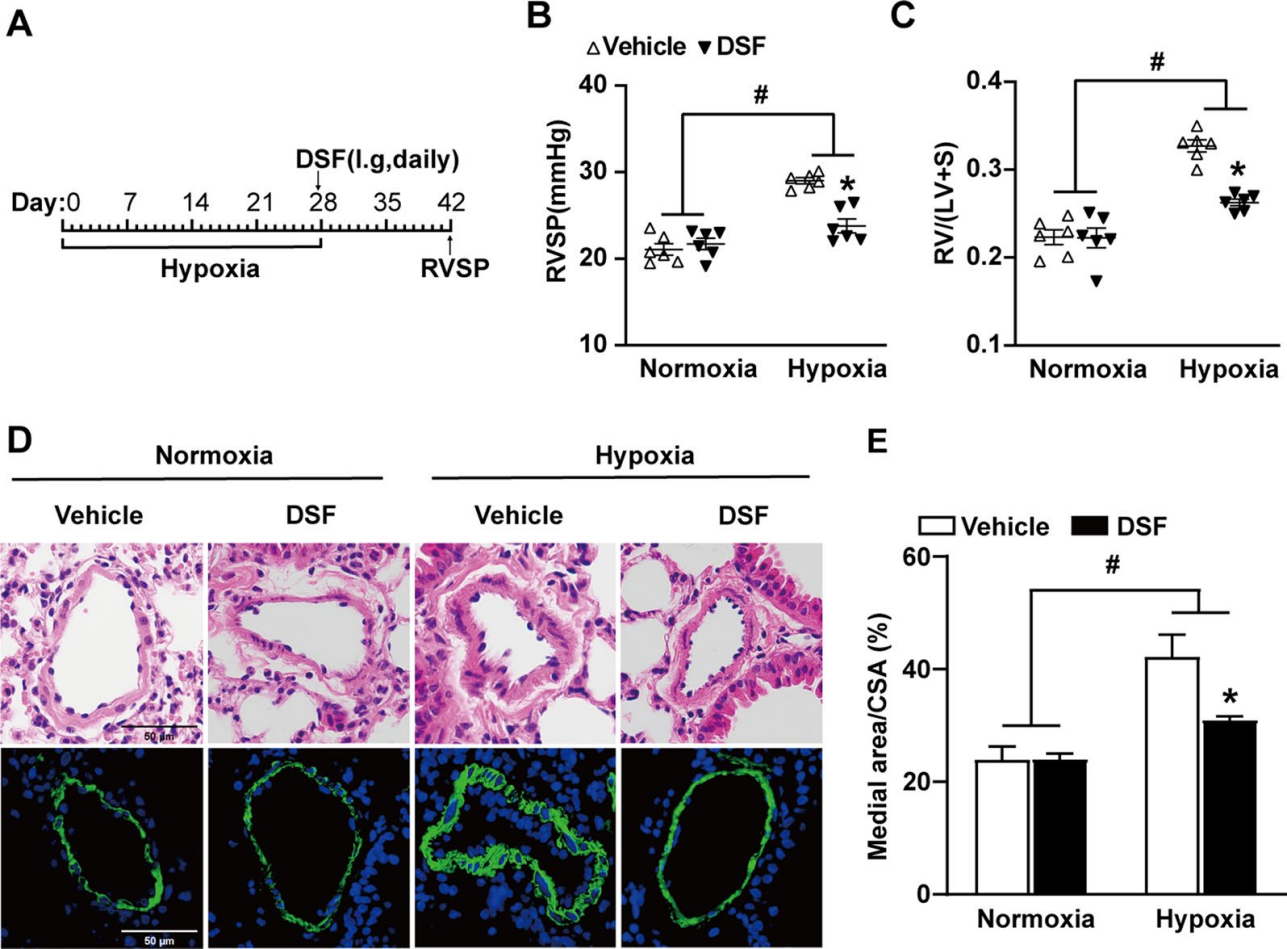
DSF attenuates the development of hypoxia-induced PH in mice. **A** Experimental design timeline of DSF therapy in hypoxia-induced PH in mice. **B**, **C** RVSP and RV/(LV + S) of mice that were treated with DSF after 4 weeks hypoxia (n = 6 each). **D** HE-stained images and immunofluorescence images of α-smooth muscle actin (green) expression in the pulmonary arteries from mice subjected to hypoxia and therapy with DSF. Scale bar: 50 μm, (n = 6 each). **E** Statistical annalysis of the ratio of medial area/CSA (pulmonary arteries with diameter 50–100 μm) for mice exposed to hypoxia and given DSF (n = 6 each). Values are the mean ± SEM. Statistical significance was analysed by one-way ANOVA test followed by Bonferroni’s multiple comparison test or the Kruskal–Wallis test. *P < 0.05 vs. vehicle, and ^#^P < 0.05 vs. normoxia (n = 5 each). i.g.: intragastric

To evaluate whether DSF elicits its action in the progression of PH, we assessed the pulmonary and right heart haemodynamic parameters as well as lung histological changes in hypoxia-induced PH mice after DSF treatment (Fig. 2A). DSF significantly attenuated PH by reducing RVSP to 82.0% (vehicle, 29.01 ± 0.35 mmHg versus DSF, 23.79 ± 0.80 mmHg; Fig. 2B) and RV/(LV + S) to 78.8% (vehicle, 0.33 ± 0.007 versus DSF, 0.26 ± 0.004; Fig. 2C). Histopathological analysis revealed that DSF treatment moderately inhibited hypoxia-induced pulmonary vascular remodelling, manifested by a reduction in medial wall thickness (%): medial area/cross-sectional area (CSA) (Fig. 2D and E).

### DSF attenuates the progression of pulmonary hypertension in rats

**Fig. 3:**
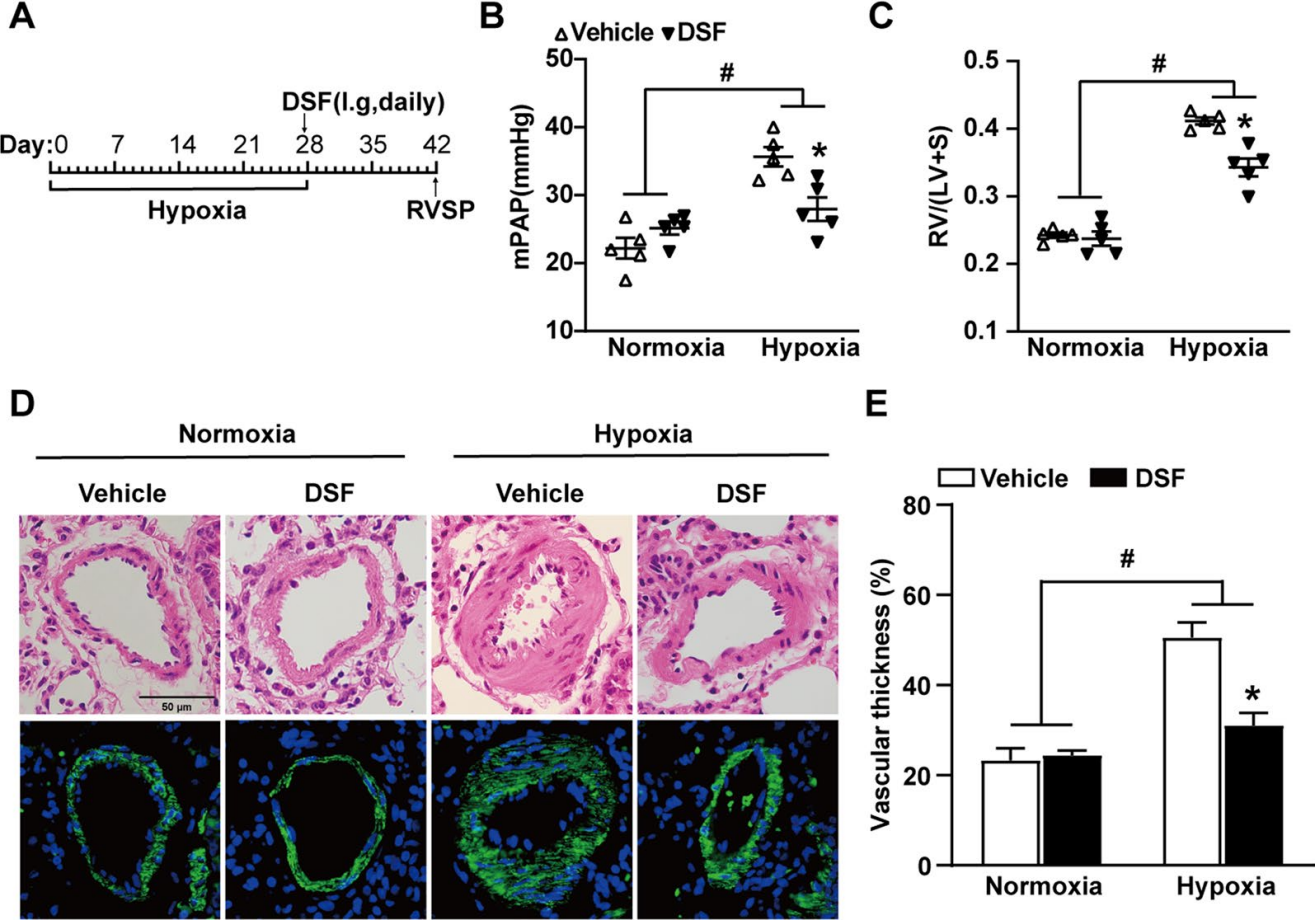
DSF attenuates the development of hypoxia-induced PH in rats. **A** Timeline of DSF therapy in hypoxia-induced PH in rats (n = 5 each). **B**, **C** RVSP and RV/(LV + S) of rats that were treated with DSF after 4 weeks of hypoxia (n = 5 each). **D** Representative HE lung sections and immunofluorescence images of α-SMA (green) in rats exposed to hypoxia and treated with DSF. Scale bar: 50 μm (n = 5 each). **E** Quantification of the medial area/CSA in hypoxia-treated rats treated with DSF (n = 5 each). Values are the mean ± SEM. Statistical significance was analysed by one-way ANOVA followed by Bonferroni’s multiple comparison test or the Kruskal–Wallis test. *P < 0.05 vs. vehicle, and ^#^P < 0.05 vs. normoxia (n = 5 each)

To evaluate the therapeutic effect of DSF in hypoixa-treated rats, oral DSF (50 mg/kg) was applied to rats after 4 weeks of hypoxia once a day (Fig. 3A). HE staining and α-SMA immunofluorescence staining showed increased artery wall thickness and a narrowed vascular lumen after 28 days of hypoxia in rats, and treatment with DSF partially reversed this vascular remodelling. Specifically, DSF markedly attenuated the increase in RVSP (DSF, 27.95 ± 1.74 mmHg versus vehicle, 35.65 ± 1.43 mmHg; P ≤ 0.001; Fig. 3B) and medial area/CSA (%) (Fig. 3D and E) in hypoxia-treated rats. In addition, rats with hypoxia-induced PH treated with DSF exhibited improvements in right heart failure, such as in a lower RV/(LV + S) (DSF, 0.34 ± 0.01 versus vehicle, 0.41 ± 0.01; P ≤ 0.001; Fig. 3C). These results indicate a reduction in the severity of PH in rats treated with DSF.

### DSF inhibits the formation of N-GSDMD and the release of IL-18 and IL-1β in the lung tissue of hypoxia-induced PH in mice and rats

**Fig. 4:**
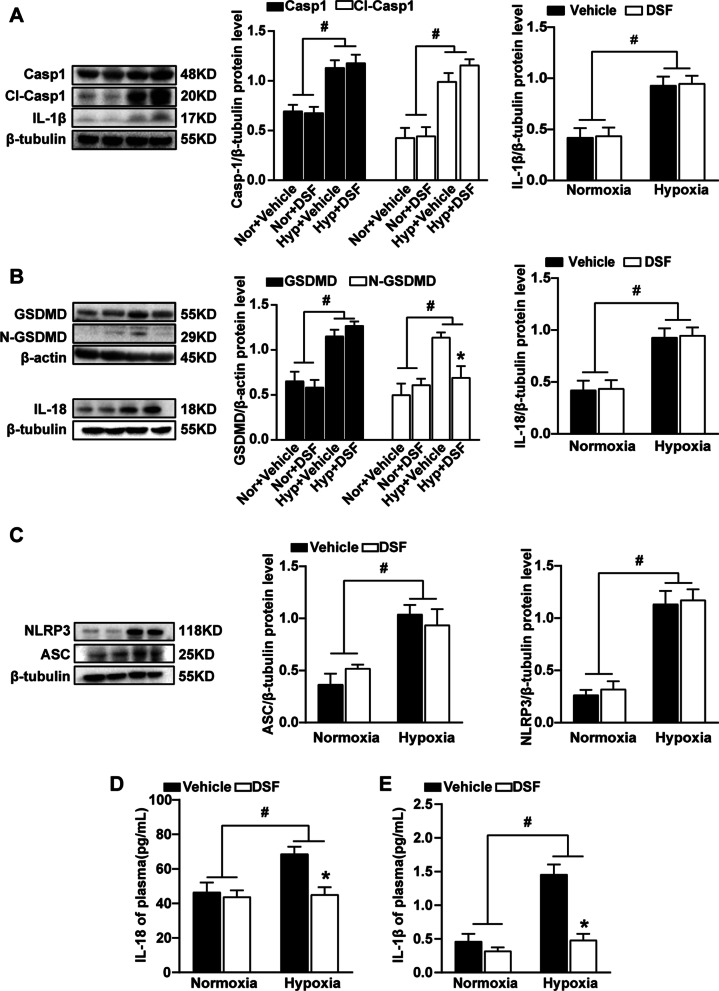
DSF inhibits the formation of N-GSDMD and the release of IL-18 and IL-1β in the lung tissue of PH mice. **A** Representative WB images and analysis of caspase-1 and CI. caspase-1 and IL-1β (n = 5 each). **B** Representative WB images and analysis of GSDMD, N-GSDMD and IL-18 (n = 5 each). **C** Representative WB images and analysis of NLRP3 and ASC. **D**, **E** ELISA detection of inflammatory factors (IL-18 and IL-1β) in the plasma, (n = 6 each). Values are the mean ± SEM. Statistical significance was analysed by one-way ANOVA followed by Bonferroni’s multiple comparison test or the Kruskal–Wallis test. *P < 0.05 vs. vehicle, and ^#^P < 0.05 vs. normoxia

**Fig. 5:**
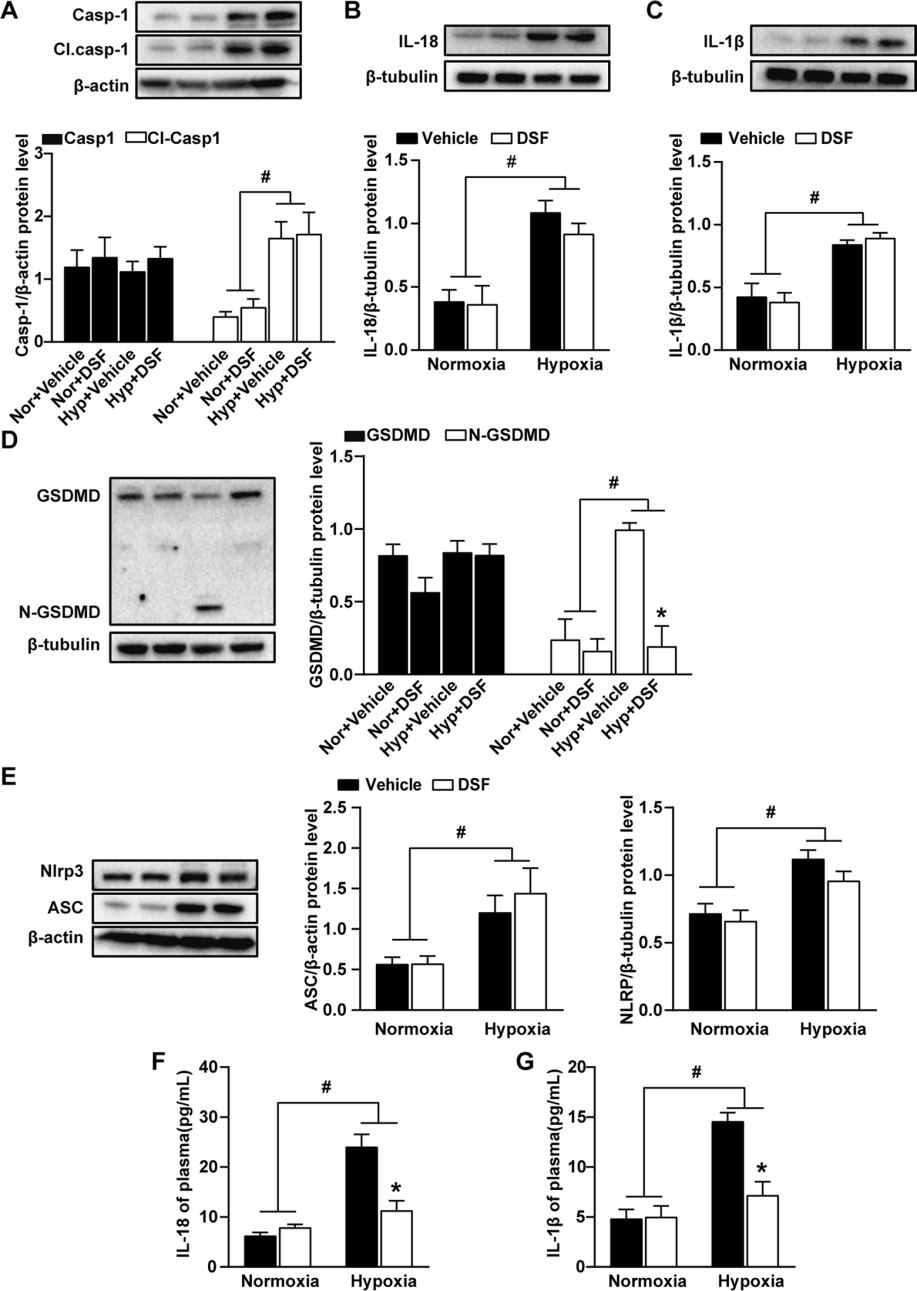
DSF inhibits the formation of N-GSDMD and the release of IL-18 and IL-1β in the lung tissue of hypoxia-induced PH rats. **A** Representative WB images and analysis of caspase-1 and CI. caspase-1. **B** Representative WB images and analysis of IL-18. **C** Representative WB images and analysis of IL-1β. **D** Representative WB images and analysis of GSDMD and N-GSDMD. **E** Representative WB images and analysis of NLRP3 and ASC. **F**, **G** ELISA detection of inflammatory factors (IL-18 and IL-1β). Values are the mean ± SEM. Statistical significance was analysed by one-way ANOVA followed by Bonferroni’s multiple comparison test or the Kruskal–Wallis test. *P < 0.05 vs. vehicle, and ^#^P < 0.05 vs. normoxia (n = 5 each)

The accumulation of N-GSDMD on the cell membrane and the formation of membrane pores promote membrane rupture that causes IL-1β and IL-18 release, which are characteristic of cell pyroptosis. Pyroptosis plays a role in the development of PH, and DSF was recently found to inhibit pyroptosis [19]. We detected the expression of canonical pyroptosis pathway-related proteins in hypoxia-treated mouse lung tissue by WB. The results showed that the expression of NLRP3, ASC, caspase1 and GSDMD in the hypoxia group was higher than that in the normoxia group (Figs. 4A–C, 5A–E). Although the expression of NLRP3, ASC, caspase1 and GSDMD showed no significant changes in the lung tissue of mice and rats exposed to hypoxia with DSF treatment, the expression of N-GSDMD was reduced. These results indicated that DSF treatment after hypoxia had no inhibitory effect on the changes in NLRP3, ASC, caspase1 and GSDMD. Furthermore, inhibiting pyroptosis reduced the release of inflammatory cytokines. Therefore, we used ELISA to detect the expression of IL-18 and IL-1β and found that the IL-18 and IL-1β concentrations in the plasma of hypoxia-treated mice and rats were significantly reduced after DSF treatment (Figs. 4D, E, 5F, G).

### DSF inhibits pyroptosis of HPASMCs under hypoxic conditions

**Fig. 6:**
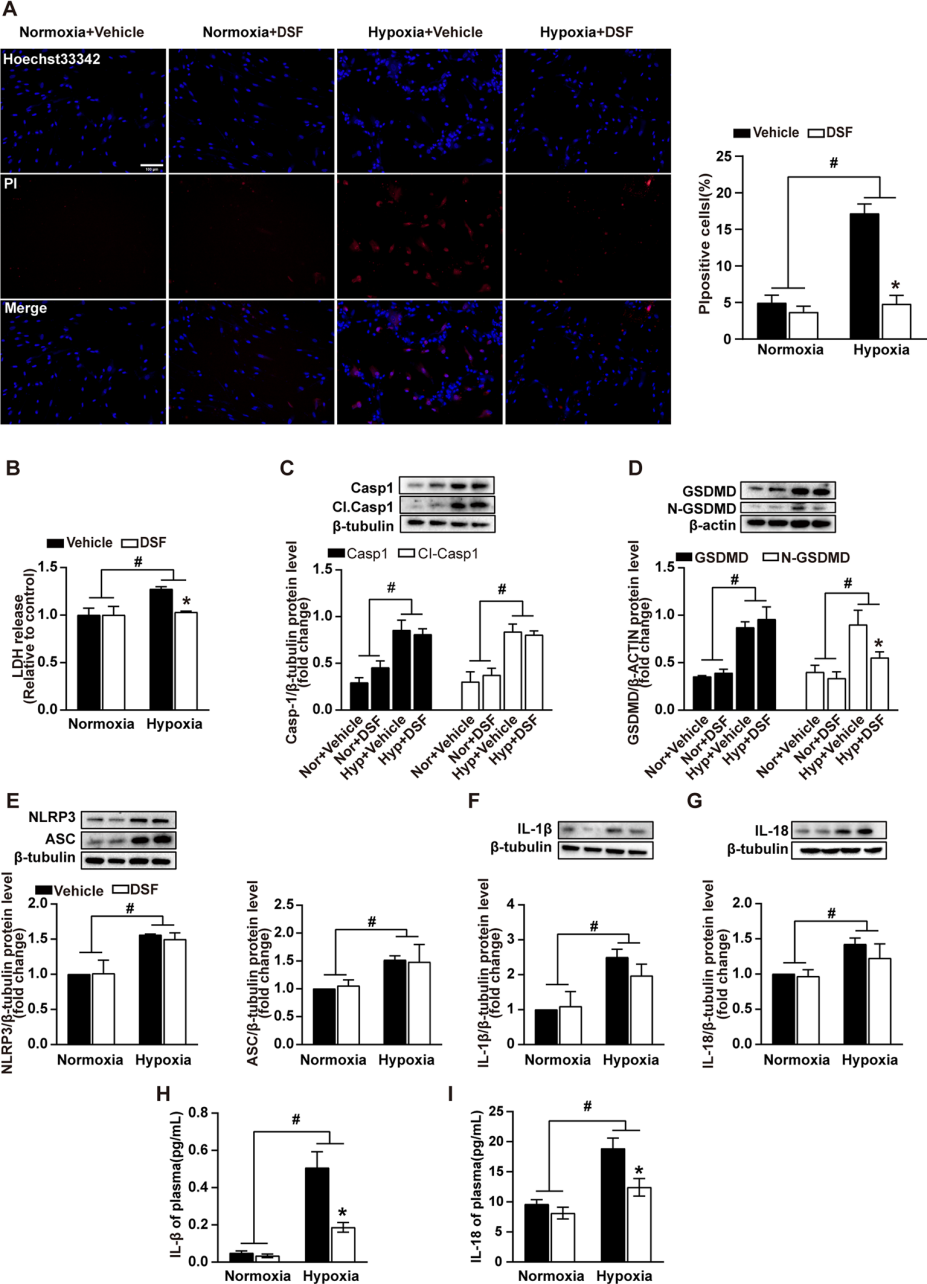
DSF inhibits pyroptosis of HPASMCs under hypoxic conditions. **A** PI staining of pyroptotic HPASMCs under hypoxic conditions was significantly reduced after DSF treatment (10 μmol/L). Red: propidium iodide (PI); Blue: Hoechst 33,342. Scale bars = 100 µm (n = 5). **B** DSF reduced the release of LDH in HPASMCs subjected to hypoxia for 24 h (n = 4). **C** Representative WB images and analysis of caspase-1 and CI. caspase-1 (n = 3). **D** Representative WB images and analysis of GSDMD and N-GSDMD (n = 3). **E** Representative WB images and analysis of NLRP3 and ASC (n = 3). **F**, **G** Representative WB images and analysis of IL-18 and IL-1β (n = 3). **H**, **I** ELISA detection of the inflammatory factors IL-1β and IL-18. Values are the mean ± SEM. Statistical significance was analysed by one-way ANOVA followed by Bonferroni’s multiple comparison test or the Kruskal–Wallis test. *P < 0.05 vs. vehicle, and ^#^P < 0.05 vs. normoxia

Inflammatory changes and immune dysregulation influence the development and progression of PH, in which cell pyroptosis plays an important role. First, the changes in pyroptosis of HPASMCs under hypoxic conditions after DSF treatment were observed. The results showed that DSF reversed hypoxia-induced pyroptosis (Fig. 6A and B). Furthermore, we continued to explore the expression of pyroptosis-related proteins in HPASMCs under hypoxic conditions after DSF treatment. The results demonstrated that DSF did not change the upregulation of NLRP3, ASC, CI.caspase1, caspase1, IL-1β, IL-18 and GSDMD were induced by hypoxia in vivo (Fig. 6B–G). However, it effectively inhibited the production of N-GSDMD, which reduced plasma membrane pore formation and cell pyroptosis (Fig. 6D). Similarly, cell culture supernatants were assayed for IL-1β and IL-18 concentrations by ELISA. The release of IL-1β and IL-18 was reduced as well (Fig. 6H and I).

## Discussion

The primary findings of this study are as follows: (a) The expression levels of lung tissue pyroptosis pathway-related genes were increased in patients with PH. (b) Plasma IL-1β and IL-18 levels in patients with PH increased. (c) DSF attenuated the development of hypoxia-induced PH in mice and rats. (d) DSF inhibited the formation of N-GSDMD and the release of IL-18 and IL-1β in the lung tissue of hypoxic mice and rats. (e) DSF inhibited HPASMC pyroptosis under hypoxic conditions. In the in vivo experiments, DSF partially reversed the progression of experimental PH, as evidenced mainly by the decreases in RVSP, mPAP, and the degree of right ventricular hypertrophy. In the in vitro experiments, DSF inhibited the cell membrane perforation and lytic death in HPASMCs.

Hypoxia is one of the most common stimuli that induce cellular pyroptosis. The combination of hypoxia and pyroptosis is involved in various diseases, such as brain injury [26, 27], cancer [28], and myocardial ischaemia/reperfusion [29, 30]. The first step in pyroptosis involves inflammasome assembly and activation. The intimate link between hypoxia and NLRP3 inflammation has been well described [31–34]. Hypoxia/reoxygenation activates NLRP3 inflammasome-mediated pyroptosis by upregulating reactive oxygen species (ROS) production [35]. Hypoxia-induced ROS contribute to myoblast pyroptosis in obstructive sleep apnoea via the NF-κB/hypoxia-inducible factor 1α (HIF-1α) signalling pathway [36]. HIF-1α may regulate inflammatory responses through the NLRP3 inflammasome complex [37, 38]. ROS and HIF-1α are involved in the development and progression of PH as the initial cellular response to hypoxic stimuli.

Both preclinical and clinical studies support the role of inflammasomes in the progression of PH [13, 39–41]. Acute and chronic inflammation responses characterize the vascular remodelling processes in PH. The initiation of inflammatory cascade plays a key role in pyroptosis and its follow-up reaction [42]. Further, most research on inflammasome activation has focused on specialized immune cells. A growing number of studies reveal that cell pyroptosis occurs in multiple cell types, including vascular smooth muscle cells (VSMCs), neutrophils, epithelial cells, dendritic cells, macrophages, endothelial cells, and cardiomyocytes [25, 43–47]. First, IL-1β receptor and IL-18 receptor are expressed at high levels on fibroblasts and VSMCs [48–50]. IL-18 and IL-1β expressed by VSMCs play important roles in cardiovascular disease [51–54]. Moreover, VSMCs can induce monocytes to express IL-1β and IL-18, which in turn promote the proliferation and migration of VSMCs [55, 56]. As mentioned earlier, both IL-18 and IL-1β can promote the proliferation and hypertrophy of HPASMCs, leading to pulmonary artery remodelling. Furthermore, inhibition of NLRP3/caspase-1/IL-1β signalling pathway can alleviate diabetic vascular remodelling [57]. GSDMD, an executor of pyroptosis, is strongly increased in patients with primary PH and in rodent PH models [58–61].

DSF, an FDA-approved drug for the treatment of alcohol addiction, has recently been repurposed as a drug for the treatment of cancer and other diseases. DSF is involved in various stress-response pathways in cells, such as antibacterial, anti-inflammatory, anti-obesity and anticancer pathways. More specifically, DSF has been leveraged to treat cancer by upregulating ROS, DNA damage, and enzymatic inhibition to inhibit the proliferation of cancer cells [62]. DSF inhibits the viability of hepatocellular carcinoma cells by disabling the HIF-mediated hypoxia signalling pathway [63]. It also inhibits N-GSDMD plasma membrane pore formation and inhibits the formation of neutrophil extracellular traps [19, 64]. These findings suggest the potential value of DSF after 60 years of research.

Pulmonary vascular remodelling is an important manifestation of the development and progression of PH, as evidenced by the hypertrophy and hyperproliferation of HPASMCs. IL-18 and IL-1β act as bridges to tightly link pyroptosis and pulmonary vascular remodelling. As described above, both IL-18 and IL-1β have a role in promoting the proliferation and hypertrophy of HPASMCs. This suggests that DSF inhibits PASMC proliferation and hypertrophy by reducing pyroptosis, which ultimately slows the process of pulmonary artery remodelling.

## Conclusion and perspectives

In this study, we found that the DSF-triggered reduction in pyroptosis in the lungs was mainly derived from reduced N-GSDMD production in HPASMCs in hypoxia-exposed mice and rats rather than from the NLRP3-ASC-caspase1-GSDMD pathway. The reduced release of IL-18 and IL-1β following DSF treatment is consistent with the downregulation of pyroptosis. This suggests that DSF exerts beneficial effects on PH by inducing reduced N-GSDMD production in HPASMCs. There is an unmet demand for therapeutic approaches concentrating on pulmonary vascular remodelling in the clinic. Our results suggest that DSF may become a therapeutic option for PH patients.

## Supplementary Information

**Additional file 1.** **Table S1.** Clinical information of PAH subjects and controls in the study. **Table S2.** Gene set enrichment analysis details.

## Data Availability

The datasets used and/or analyzed during the current study are available from the corresponding author on reasonable request.

## Abbreviations

PH: Pulmonary hypertension
DSF: Disulfiram
GSDMD: Gasdermin D
N-GSDMD: N-terminal domain of GSDMD
Cl.Caspase: Cleaved-caspase
RVSP: Right ventricular systolic pressure
mPAP: Mean pulmonary artery pressure
RVH: Right ventricular hypertrophy
HPASMCs: Human pulmonary artery smooth muscle cells
IL: Interleukin
NLRP3: Nucleotide-binding oligomerization domain-like protein3
ASC: Apoptosis-associated speck-like protein containing a caspase-recruitment domain
LDH: Lactate dehydrogenase
GSEA: Gene set enrichment analysis
NES: Normalized enrichment score
ELISA: Enzyme-linked immunosorbent assay
WB: Western blot
VSMCs: Vascular smooth muscle cells
