# Supplementary material for: Disulfiram attenuates hypoxia-induced pulmonary hypertension by inhibiting GSDMD cleavage and pyroptosis in HPASMCs
Source: Respir Res. 2022 Dec 16;23:353. doi: 10.1186/s12931-022-02279-0 (PMC9756478; doi:10.1186/s12931-022-02279-0)
Supplement: Supplementary file 1 — Additional file 1. Table S1. Clinical information of PAH subjects and controls in the study. Table S2. Gene set enrichment analysis details. [file 12931_2022_2279_MOESM1_ESM.pdf]

Table S1. Clinical information of PAH subjects and controls in the study

Patient	Gender	Age, y	Condition	Right Heart Catheterization (mmHg)					PVR	SVR
				CVP	RAP	RVP	mPAP	mPAWP		
1	Male	77	Control	7	7	8	17	21	13	3.1
2	Female	70	Control	9	9	10	13	22	8	2.91
3	Male	60	PAH	27	27	27	37	42	32	2.07
4	Female	78	Control	7	**7**	8	14	19	9	2.9
5	Male	68	Control	5	5	5	13	16	11	3.12
6	Male	79	PAH	9	9	10	20	25	15	3.1
7	Female	45	PAH	22	22	23	47	54	19	3.61
8	Male	59	Control	17	17	15	31	40	29	3.34
9	Male	76	PAH	10	10	8	14	21	14	2.72
10	Female	69	Control	7	7	6	12	19	7	2.97
11	Male	49	PAH	12	12	15	21	21	15	NA
12	Male	82	Control	5	5	5	12	16	12	3.38
13	Male	67	Control	1	1	1	5	9	4	3.05
14	Male	57	Control	5	5	4	14	16	14	4.17
15	Male	75	PAH	9	9	8	21	26	14	3.12
16	Female	61	Control	2	2	2	NA	18	8	4.24
17	Male	57	PAH	9	9	11	17	22	15	3.13
18	Female	57	Control	6	6	7	13	16	9	2
19	Male	34	PAH	14	14	11	26	33	19	3.16
20	Female	53	PAH	6	6	8	20	28	9	3.14
21	Female	60	PAH	5	5	5	18	27	12	3.31
22	Female	38	Control	7	7	8	13	16	12	2.9
23	Female	53	PAH	4	4	NA	NA	36	NA	NA
24	Female	66	PAH	5	5	NA	NA	60	12	2.01
25	Female	59	Control	10	10	NA	8	18	14	2.8
26	Male	56	PAH	7	7	4	18	26	9	4.01
27	Female	74	Control	6	6	NA	NA	18	12	3.24
28	Male	81	Control	10	10	NA	21	21	12	3.44
29	Male	64	Control	3	3	NA	NA	15	11	5.04
30	Male	58	PAH	8	8	10	NA	28	NA	NA

Abbreviations:CVP,central venous pressure;RAP, right atrial pressure; RVP, right ventricular pressure; mPAP; mean pulmonary artery pressure; mPAWP, mean pulmonary capillary wedge pressure; PVR,pulmonary vascular resistance; SVR, systemic vascular resistance.

Table S2: Gene set enrichment analysis details

Gene symbol	Rank in gene list	Rank metric score	Running ES	Core enrichment
MMP1	162	0.303	0.0481	Yes
AIM2	238	0.272	0.0955	Yes
CHMP4C	266	0.266	0.1445	Yes
CASP8	275	0.264	0.1943	Yes
NLRC4	281	0.262	0.244	Yes
CHMP2B	709	0.193	0.2554	Yes
NLRP3	844	0.18	0.2817	Yes
GBP3	922	0.172	0.31	Yes
HMGB1	945	0.171	0.3412	Yes
GBP5	950	0.171	0.3735	Yes
GBP2	1281	0.148	0.382	Yes
TLR5	1347	0.144	0.4057	Yes
GBP1	1475	0.137	0.4243	Yes
IL1A	1611	0.13	0.4411	Yes
MEFV	1722	0.125	0.4583	Yes
BTK	1760	0.123	0.4796	Yes
GBP4	1800	0.121	0.5004	Yes
CARD18	1939	0.116	0.5142	Yes
IL1B	2021	0.112	0.5307	Yes
VSIG4	2228	0.104	0.5382	Yes
PYDC1	2858	0.086	0.5171	Yes
TLR8	3040	0.082	0.5219	Yes
TLR1	3093	0.081	0.5342	Yes
CASP4	3621	0.069	0.5159	Yes
CASP3	3814	0.065	0.5169	Yes
MMP9	3855	0.064	0.5268	Yes
CHMP3	4129	0.059	0.5219	Yes
TLR2	4209	0.058	0.5282	Yes
IL18	4285	0.056	0.5345	Yes
DDX3X	4438	0.054	0.5358	Yes
NLRP2	4568	0.052	0.538	Yes
TLR4	4792	0.048	0.5339	Yes
TREM2	4846	0.048	0.5398	Yes
DHX9	4902	0.047	0.5455	Yes
TLR6	4953	0.046	0.5513	Yes
IRF2	5138	0.043	0.5486	No
GSDMC	5295	0.041	0.547	No

CASP1	5420	0.039	0.5471	No
TLR3	5576	0.037	0.5449	No
CHMP2A	5897	0.033	0.5321	No
TXNIP	6642	0.023	0.4921	No
CHMP7	6645	0.022	0.4963	No
BAK1	6940	0.019	0.4824	No
TLR9	7098	0.017	0.4763	No
CTSG	7164	0.016	0.4756	No
CHMP6	7878	0.008	0.4346	No
CFLAR	8087	0.005	0.4233	No
PYCARD	8097	0.005	0.4237	No
CASP6	8099	0.005	0.4246	No
CASP5	8100	0.005	0.4256	No
TNFRSF21	8264	0.003	0.4165	No
CHMP4A	8553	0	0.3994	No
ZBP1	8581	0	0.3978	No
CYCS	8676	-0.001	0.3925	No
GSDMD	9188	-0.007	0.3634	No
IRF1	9936	-0.015	0.3219	No
NLRP12	10121	-0.017	0.3143	No
CARD8	10207	-0.018	0.3127	No
BAX	10343	-0.02	0.3085	No
CHMP4B	10388	-0.021	0.3098	No
APIP	10591	-0.023	0.3021	No
NAIP	10904	-0.027	0.2886	No
SERPINB1	11432	-0.033	0.2635	No
CARD16	12140	-0.042	0.2295	No
GSDMA	14586	-0.09	0.101	No
GSDMB	14620	-0.091	0.1163	No
NLRP7	14643	-0.091	0.1324	No
